# Association between WNT-1-inducible signaling pathway protein-1 (WISP1) genetic polymorphisms and the risk of gastric cancer in Guangxi Chinese

**DOI:** 10.1186/s12935-021-02116-2

**Published:** 2021-07-30

**Authors:** Yanqiong Liu, Weijuan Qin, Fuyong Zhang, Jian Wang, Xi Li, Shan Li, Xue Qin, Yuefeng Lu

**Affiliations:** 1 Department of Clinical Laboratory, The First Affiliated Hospital of Guangxi Medical University, Nanning, Guangxi China; 2 Department of Clinical Laboratory, The Second Affiliated Hospital of Guangxi Medical University, Nanning, Guangxi China; 3 Medical Equipment Department, The First Affiliated Hospital of Guangxi Medical University, Nanning, Guangxi China

**Keywords:** WISP1, Polymorphism, Gastric cancer, Risk

## Abstract

**Background:**

WNT1-inducible signaling pathway protein 1 (WISP1) is a member of the CCN protein family and a downstream target of β-catenin. Aberrant WISP1 expression may be involved in carcinogenesis. To date, no studies have investigated the association between single-nucleotide polymorphisms (SNPs) of WISP1 and gastric cancer. Therefore, we conducted this study to explore their relationship.

**Methods:**

Polymerase chain reaction-restriction fragment length polymorphism assay was used to analyze three SNPs of WISP1 in 204 gastric cancer patients and 227 controls.

**Results:**

Overall, we could not identify a significant association between WISP1 SNPs and gastric cancer risk. However, the subgroup analysis demonstrated that the presence of the rs7843546 T allele was associated with a significantly decreased risk of gastric cancer in those of Han Chinese ethnicity (CT vs. CC: OR = 0.33, 95%CI 0.14–0.78; TT vs. CC: OR = 0.29, 95%CI 0.11–0.76; CT + TT vs. CC: OR = 0.32, 95%CI 0.14–0.74). In addition, patients with the rs7843546 TT genotype display a 0.34-fold lower risk of developing stage I/II gastric cancer than those with the CC genotype Furthermore, individuals ≥ 50 years old who carried the rs10956697 AC genotype had a significantly decreased risk of gastric cancer (OR = 0.58, 95%CI 0.35–0.98). Smokers with the rs10956697 AC and AC + AA genotypes exhibited a 0.28-fold lower and 0.32-fold lower risk of gastric cancer, respectively.

**Conclusions:**

The WISP1 SNPs rs7843546 and rs10956697 were, for the first time, found to reduce susceptibility to gastric cancer in various subgroups of Guangxi Chinese.

**Supplementary Information:**

The online version contains supplementary material available at 10.1186/s12935-021-02116-2.

## Background

Globally, the burden of cancer incidence and mortality is rapidly growing. According to estimates from the World Health Organization (WHO), gastric cancer remains a commonly cancer worldwide and is responsible for 1,089,103 new cases and an estimated 768,793 deaths in 2020, ranking fifth for incidence and fourth for mortality globally [1]. China is a high-incidence region in terms of gastric cancer. According to the 2020 global cancer statistics, 478,508 new gastric cancer cases and 373,789 deaths were estimated to have occurred in China in 2020, accounting for 43.9% and 48.6% of cases and deaths worldwide, respectively [2]. The high incidence and mortality in China highlight the importance of understanding the risk factors related to gastric cancer development. Also, gastric cancer has a multifactorial etiology in terms of risk factors, carcinogenesis, and epidemiologic patterns [3]. Chronic *Helicobacter pylori* infection is considered the primary cause, with almost all cases attributed to this bacterium [4]. The prevalence of *H. pylori* infection is extremely high, infecting half of the world’s population [5]. However, only about 1% people with *Helicobacter* infections will develop gastric cancer, likely because of differences in host genetics, gender, age of infection acquisition, alcohol consumption, tobacco smoking, and environmental factors [6]. More novel and powerful methods of identifying the predisposing genetic factors are expected to provide new insights regarding the basic molecular pathways involved in tumorigenesis.

WNT1-inducible signaling pathway protein-1 (WISP1), also known as CCN4, is a cysteine-rich protein that belongs to the CCN protein family [7]. WISP1 is a target of the Wnt1 pathway, which can modulate multiple processes that involve tumorigenesis and stem cell proliferation [8]. WISP1 aberrant expression is associated with the promotion of various pathologies, including osteoarthritis, fibrosis, and cancer [9]. In 2017, Jia et al. first suggested that WISP1was up-regulated in gastric cancer and acted as an oncogene by promoting proliferation, migration, and invasion in gastric cancer cells [10]. Additionally, Zhang et al. demonstrated that significantly up-regulated WISP1 expression was associated with cancer progression, chemotherapy outcomes, and poor prognosis in gastric cancer in 2019 [11]. The observation that WISP1 plays an important role in the progression of gastric cancer highlights the importance of identifying the variants of this gene because a single nucleotide polymorphism (SNP) can change the encoded amino acids in a protein when it arises in the related coding sequence, thus influencing gene function and phenotype [12].

The WISP1 gene consists of five exons and four introns located on chromosome 8q24.1 to 8q24.3, and it has been shown to be highly polymorphic [13]. Several clinical studies have indicated a significant association between WISP1 polymorphisms and various cancers, such as breast cancer [14], urothelial cell carcinoma [15], hepatocellular carcinoma [16], oral squamous cell carcinoma [17], lung cancer [18] and uterine cervical cancer [19]. Up until now, to our knowledge, no study has established a connection between WISP1 genetic polymorphisms and gastric cancer. Therefore, we conducted this study to explore the association between the WISP1 SNPs rs2929973, rs7843546, and rs10956697 and susceptibility to gastric cancer in a Guangxi (Southwest China) population.

## Methods

### Sample size consideration

We estimated the sample size using Quanto software (Version 1.2.4) [20, 21]. An unmatched case–control design will be used. The prevalence of the WISP1 SNP rs2929973 T allele in the HapMap global population is 0.8451 (HapMap Project dbSNP database: http://www.ncbi.nlm.nih.gov/snp/). The inheritance model is recessive. The relative risk is 2.0. The desired power is 80% at a significance level of 0.05, with a two-sided alternate hypothesis. According to the above parameters, the estimated 186 case–control pairs required for the desired power to assess the risk of WISP1 genetic variation on gastric cancer development.

#### Study subjects

We enrolled 204 gastric cancer patients in this study. All cases were newly clinically and pathologically confirmed as primary gastric cancer without a history of abdominal surgery and admitted to the First Affiliated Hospital of Guangxi Medical University, Guangxi, China, as has been described in our previous study [22]. Patients were excluded if they had any of the following: (a) concomitant malignant neoplasia, (b) acquired immunodeficiency syndrome, (c) acute or chronic inflammatory diseases, and (d) positive antibodies for *H. pylori*.

For the control group, we selected 227 healthy individuals recruited from the general health check-up centers at the same hospital during the same period of the study. The individuals in the control group had no previous genetic history of the tumor and were matched with the case group in terms of gender and age. The clinical and pathological characteristics of all subjects were collected based on an electronic medical record system.

### Selection of WISP1 SNPs

For this study, SNPs were selected based on data from the International HapMap Project (http://hapmap.ncbi.nlm.nih.gov/), dbSNP database (http://www.ncbi.nlm.nih.gov/projects/SNP/), and findings of previous studies reporting the effect of WISP1 genetic polymorphisms on cancer susceptibility [14–19, 23]. All SNPs had minor allele frequencies (MAF) of > 5% to prevent false-negative results. Based on the aforementioned criteria, three SNPs were selected: rs2929973, rs7843546, and rs10956697.

### DNA extraction and WISP1 genotyping

Genomic DNA was isolated from EDTA-anticoagulated venous blood using the phenol–chloroform protocol, as described in detail in our previous studies [22, 24]. The concentration and purity of the DNA were determined spectrophotometrically. The obtained DNA was stored at − 20 °C and prepared for genotyping using polymerase chain reaction (PCR). Genotyping was conducted via restriction fragment length polymorphism (RFLP) assay, as described previously [22, 24].

### DNA sequencing

To determine the accuracy of the polymerase chain reaction-restriction fragment length polymorphism (PCR-RFLP) method, a random selection of > 5% of all samples was genotyped via the direct sequencing method with an ABI Prism 3100 (Applied Biosystems, Shanghai Sangon Biological Engineering Technology & Services Co., Ltd., China). The resultant genotypes showed no differences.

### Statistical analysis

A student’s *T*-test or Mann–Whitney *U* test was applied to analyze the continuous variables. The χ^2^ test or Fisher’s exact test was applied to analyze the categorical variables. Adjusted odds ratios (AORs) and 95% confidence intervals (CIs) were estimated using logistic regression models. These AORs and 95%CIs were used to assess the association between genotype frequencies and gastric cancer risk and clinical and pathological characteristics. To evaluate the joint effects of the three SNPs in the WISP1 gene, SHEsis software (http://analysis.bio-x.cn/myAnalysis.php) [25] was employed to construct haplotypes between the patients and controls. SPSS Version 16.0 for Windows (SPSS Inc., IL, USA) software was used for all the statistical analyses. A two-sided P*-*value of < 0.05 was accepted as statistically significant.

## Results

### Characteristics of the study subjects

Table 1 shows the demographic and clinical characteristics of all the subjects in the study. In total, 204 gastric cancer patients and 227 controls were enrolled in the study. Their ages and sexes were well-matched (P = 0.057 and P = 0.954, respectively). There were no differences between the two groups in terms of smoking, drinking alcohol, or ethnicity. The patient groups had a significantly lower average BMI as compared to the healthy controls. Most patients (70.6%) had stage III/IV gastric cancer, while 29.4% had stage I/II disease. Most tumors (82.4%) were classified as undifferentiated or poorly differentiated (Table 1).

**Table 1:** The distributions of demographical characteristics in 227 controls and 204 patients with gastric cancer

Characteristics	Patients (N = 204)	Controls (N = 227)	*P* value
Ages(mean ± SD, years)	54.31 ± 12.00	52.58 ± 5.13	0.057
BMI (mean ± SD, kg/m^2^)	20.59 ± 3.11	22.47 ± 3.47	< 0.001^*^
Gender			
Male	134 (65.7%)	138 (60.8%)	0.954
Female	70 (34.0%)	89 (39.2%)	
Ethnicity			
Han	99 (48.5%)	112 (49.3%)	0.975
Zhuang	92 (45.1%)	100 (44.1%)	
Other	13 (6.4%)	15 (6.6%)	
Smoking			
Yes	58 (28.4%)	71 (31.3%)	0.519
No	146 (71.6%)	156 (68.7%)	
Drinking alcohol			
Yes	52 (25.5%)	67 (29.5%)	0.351
No	152 (74.5%)	160 (70.5%)	
Cell differentiation			
Moderate and poor	168 (82.4%)		
Well	36 (17.6%)		
Clinical stage			
I/II	60 (29.4%)		
III/IV	144 (70.6%)		
Family history of gastric cancer			
Yes	6 (2.9)	4 (1.8)	0.833
No	198 (97.1)	223 (98.2)	

*SD* standard deviation

^*^ P value < 0.05 as statistically significant

### WISP1 polymorphisms and gastric cancer risk

The genotypic distributions of the WISP1 SNPs rs2929973, rs7843546, and rs10956697 in the gastric cancer group and normal control group were all in accordance with the Hardy–Weinberg equilibrium (P > 0.05). The frequency distribution and logistic regression analysis of the polymorphisms of the WISP1 gene in the gastric cancer and control groups are shown in Table 2. After logistic regression adjustment analysis based on gender, age, BMI, ethnicity, smoking, and drinking alcohol, no significant differences were observed between gastric cancer patients and the control group in terms of the rs2929973, rs7843546, and rs10956697 polymorphisms of the WISP1 gene.

**Table 2:** The frequency distribution and logistic regression analysis of the polymorphism of WISP1 gene in gastric cancer and control group

Variables	Gastric cancer (N = 204) n (%)	Controls (N = 227) n (%)	AOR (95% CI)	*P*
rs2929973				
Alleles				
T	285 (69.9)	295 (65.0)	1.00^ref^	
G	123 (30.1)	159 (35.0)	0.83 (0.61–1.13)	0.230
Co-dominant				
TT	100 (49.0)	102 (44.9)	1.00^ref^	
TG	85 (41.7)	91 (40.1)	0.99 (0.64–1.54)	0.967
GG	19 (9.3)	34 (15.0)	0.63 (0.32–1.21)	0.164
Dominant				
TT	100 (49.0)	102 (44.9)	1.00^ref^	
TG + GG	104 (51.0)	125 (55.1)	0.88 (0.58–1.32)	0.536
Recessive				
TT + TG	185 (90.7)	193 (85.0)	1.00^ref^	
GG	19 (9.3)	34 (15.0)	0.62 (0.33–1.17)	0.141
rs7843546				
Allele				
C	191 (46.8)	191 (42.1)	1.00^ref^	
T	217 (53.2)	263 (57.9)	0.84 (0.63–1.12)	0.224
Co-dominant				
CC	41 (20.1)	36 (15.9)	1.00^ref^	
CT	109 (53.4)	119 (52.4)	0.86 (0.49–1.48)	0.577
TT	54 (26.5)	72 (31.7)	0.68 (0.37–1.24)	0.204
Dominant				
CC	41 (20.1)	36 (15.9)	1.00^ref^	
CT + TT	163 (79.9)	191 (84.1)	0.79 (0.47–1.33)	0.376
Recessive				
CT + CC	150 (73.5)	155 (68.3)	1.00^ref^	
TT	54 (26.5)	72 (31.7)	0.78 (0.50–1.21)	0.262
rs10956697				
Allele				
C	278 (68.1)	286 (63.0)	1.00^ref^	
A	130 (31.9)	168 (37.0)	0.88 (0.65–1.20)	0.421
Co-dominant				
CC	95 (46.6)	86 (37.9)	1.00^ref^	
AC	88 (43.1)	114 (50.2)	0.78 (0.50–1.19)	0.248
AA	21 (10.3)	27 (11.9)	0.90 (0.45–1.79)	0.771
Dominant				
CC	95 (46.6)	86 (37.9)	1.00^ref^	
AC + AA	109 (53.4)	141 (62.1)	0.79 (0.52–1.19)	0.254
Recessive				
CC + AC	183 (89.7)	200 (88.1)	1.00^ref^	
AA	21 (10.3)	27 (11.9)	1.02 (0.53–1.96)	0.947

*ref* reference; *AOR* Adjusted odds ratio; *95% CI* 95% confidence interval; adjusted for age, BMI ethnicity, smoking and drinking alcohol

To clarify the role of WISP1 genetic polymorphisms in gastric cancer’s demographic and clinical variables, the respective SNPs were analyzed to determine their correlations with clinical parameters. Tables 3, 4, 5 and Additional files 1, 2, 3, 4 present the results of the subgroup analyses performed by clinical stage, cell differentiation, gender, age, ethnicity, smoking status, and drinking status. In an evaluation of clinical stage and rs7843546 WISP1 genotypes, patients with the TT genotype displayed a 0.34-fold lower risk of developing stage I/II gastric cancer than those with the CC genotype (OR = 0.34; 95% CI 0.14–0.84; P = 0.020) (Table 3) after adjusting for gender, age, BMI, ethnicity, smoking, and drinking. In addition, after adjustment for the above-mentioned variables, subjects carrying at least one copy of the T allele for the rs7843546 SNP exhibited a 0.46-fold lower risk of stage I/II gastric cancer than those with the CC genotype (dominant model: CT + TT vs. CC, OR = 0.46, 95% CI 0.22–0.96, P = 0.038) (Table 3). However, the other SNPs genotypes did not exhibit a significant difference in this regard.

**Table 3:** Distribution frequency of WISP1polymorphisms in controls and gastric cancer patients stratified by clinical stage

Variables	Clinical stage I/II				Clinical stage III/IV		
	Controls (N = 227)	Cancer (N = 60)	*OR (95% CI)	*P*	Cancer (N = 144)	AOR (95% CI)	*P*
rs2929973							
Co-dominant TT	102 (44.9)	26 (43.3)	1.00^ref^		74 (51.4)	1.00^ref^	
TG	91 (40.1)	28 (46.7)	1.30 (0.46–3.70)	0.624	57 (39.6)	0.88 (0.54–1.42)	0.591
GG	34 (15.0)	6 (10.0)	1.71 (0.60–4.85)	0.315	13 (9.0)	0.58 (0.28–1.21)	0.144
Dominant TT	102 (44.9)	26 (43.3)	1.00^ref^		74 (51.4)	1.00^ref^	
TG + GG	125 (55.1)	34 (56.7)	1.17 (0.62–2.20)	0.636	70 (48.6)	0.78 (0.50–1.23)	0.286
Recessive TT + TG	193 (85.0)	54 (90.0)	1.00^ref^		131 (91.0)	1.00^ref^	
GG	34 (15.0)	6 (10.0)	0.67 (0.25–1.81)	0.433	13 (9.0)	0.61 (0.30–1.23)	0.163
rs7843546							
Co-dominant CC	36 (15.9)	17 (28.3)	1.00^ref^		24 (16.7)	1.00^ref^	
CT	119 (52.4)	30 (50.0)	0.54 (0.25–1.17)	0.116	79 (54.9)	1.08 (0.58–2.02)	0.808
TT	72 (31.7)	13 (21.7)	0.34 (0.14–0.84)	0.020^*^	41 (28.5)	0.90 (0.46–1.77)	0.756
Dominant CC	36 (15.9)	17 (28.3)	1.00^ref^		24 (16.7)	1.00^ref^	
CT + TT	191 (84.1)	43 (71.7)	0.46 (0.22–0.96)	0.038^*^	120 (83.3)	1.02 (0.56–1.85)	0.953
Recessive CT + CC	155 (68.3)	47 (78.3)	1.00^ref^		103 (71.5)	1.00^ref^	
TT	72 (31.7)	13 (21.7)	0.52 (0.25–1.10)	0.087	41 (28.5)	0.87 (0.54–1.41)	0.569
rs10956697							
Co-dominant CC	86 (37.9)	26 (43.3)	1.00^ref^		69 (47.9)	1.00^ref^	
AC	114 (50.2)	27 (45.0)	0.84 (0.43–1.63)	0.609	61 (42.4)	0.72 (0.45–1.16)	0.176
AA	27 (11.9)	7 (11.7)	1.08 (0.38–3.03)	0.888	14 (9.7)	0.83 (0.39–1.79)	0.636
Dominant CC	86 (37.9)	26 (43.3)	1.00^ref^		69 (47.9)	1.00^ref^	
AC + AA	141 (62.1)	34 (56.7)	0.88 (0.47–1.66)	0.696	75 (52.1)	0.73 (0.46–1.14)	0.166
Recessive CC + AC	200(88.1)	53 (88.3)	1.00^ref^		130 (90.3)	1.00^ref^	
AA	27 (11.9)	7 (11.7)	1.18 (0.45–3.13)	0.739	14 (9.7)	0.97 (0.47–2.01)	0.943

*ref* reference; *AOR* Adjusted odds ratio; *95% CI* 95% confidence interval; adjusted for age, BMI ethnicity, smoking and drinking alcohol

* P < 0.05 as statically significant

**Table 4:** Distribution frequency of WISP1polymorphisms in controls and gastric cancer patients stratified by ethnicity

Variables	Han				Zhuang			
	Cancer (N = 99)	Controls (N = 112)	OR (95% CI)	*P*	Cancer (N = 92)	Controls (N = 100)	OR (95% CI)	*P*
rs2929973								
Co-dominant TT	50	56	1.00^ref^		45	40	1.00^ref^	
TG	38	40	1.19(0.62–2.29)	0.601	40	42	0.90(0.46–1.74)	0.750
GG	11	16	0.93(0.37–2.36)	0.875	7	18	0.36(0.13–1.02)	0.054
Dominant TT	50	56	1.00^ref^		45	40	1.00^ref^	
TG + GG	49	56	1.08(0.59–1.96)	0.801	47	60	0.73(0.39–1.36)	0.324
Recessive TT + TG	88	96	1.00^ref^		85	82	1.00^ref^	
GG	11	16	0.85(0.35–2.06)	0.714	7	18	0.38(0.14–1.02)	0.055
rs7843546								
Co-dominant CC	23	14	1.00^ref^		15	17	1.00^ref^	
CT	50	61	0.33(0.14–0.78)	0.012^*^	52	51	1.53(0.64–3.64)	0.335
TT	26	37	0.29(0.11–0.76)	0.012^*^	25	32	0.94(0.37–2.38)	0.896
Dominant CC	23	14	1.00^ref^		15	17	1.00^ref^	
CT + TT	76	98	0.32(0.14–0.74)	0.007^*^	77	83	1.27(0.56–2.87)	0.570
Recessive CT + CC	73	75	1.00^ref^		67	68	1.00^ref^	
TT	26	37	0.73(0.37–1.41)	0.348	25	32	0.69(0.35–1.36)	0.285
rs10956697								
Co-dominant CC	48	41	1.00^ref^		41	36	1.00^ref^	
AC	38	58	0.71(0.37–1.36)	0.308	44	50	0.82(0.43–1.56)	0.542
AA	13	13	1.23(0.47–3.23)	0.678	7	14	0.52(0.18–1.56)	0.246
Dominant CC	48	41	1.00^ref^		41	36	1.00^ref^	
AC + AA	51	71	0.78(0.42–1.42)	0.412	51	64	0.76(0.41–1.42)	0.385
Recessive CC + AC	86	99	1.00^ref^		85	86	1.00^ref^	
AA	13	13	1.42(0.57–3.51)	0.449	7	14	0.59(0.21–1.65)	0.310

*ref* reference; *AOR* Adjusted odds ratio; *95% CI* 95% confidence interval; adjusted for gender, age, BMI smoking and drinking alcohol; * *P* < 0.05 as statically significant

**Table 5:** Distribution frequency of WISP1polymorphisms in controls and gastric cancer patients stratified by cell differentiation

Variables	Moderate and poor differentiation				Well differentiation		
	Controls (N = 227)	Cancer (N = 168)	AOR (95% CI)	*P*	Cancer (N = 36)	AOR (95% CI)	*P*
rs2929973							
Co-dominant TT	102(44.9)	81(48.2)	1.00^ref^		19(52.8)	1.00^ref^	
TG	91(40.1)	72(42.9)	1.03(0.65–1.62)	0.912	13(36.1)	0.86(0.36–2.07)	0.730
GG	34(15.0)	15(8.9)	0.60(0.29–1.21)	0.151	4(11.1)	0.96(0.27–3.39)	0.953
Dominant TT	102(44.9)	81(48.2)	1.00^ref^		19(52.8)	1.00^ref^	
TG + GG	125(55.1)	87(51.8)	0.90(0.58–1.37)	0.612	17(47.2)	0.88(0.39–1.99)	0.766
Recessive TT + TG	193(85.0)	153(91.1)	1.00^ref^		32(88.9)	1.00^ref^	
GG	34(15.0)	15(8.9)	0.59(0.30–1.14)	0.117	4(11.1)	1.03(0.31–3.42)	0.960
rs7843546							
Co-dominant CC	36(15.9)	34(20.2)	1.00^ref^		7(19.4)	1.00^ref^	
CT	119(52.4)	86(51.2)	0.79(0.45–1.41)	0.426	23(63.9)	1.14(0.40–3.24)	0.801
TT	72(31.7)	48(28.6)	0.71(0.38–1.31)	0.272	6(16.7)	0.42(0.11–1.56)	0.197
Dominant CC	36(15.9)	34(20.2)	1.00^ref^		7(19.4)	1.00^ref^	
CT + TT	191(84.1)	134(79.8)	0.77(0.45–1.32)	0.333	29(80.6)	0.87(0.31–2.39)	0.782
Recessive CT + CC	155(68.3)	120(71.4)	1.00^ref^		30(83.3)	1.00^ref^	
TT	72(31.7)	48(28.6)	0.86(0.54–1.35)	0.504	6(16.7)	0.38(0.14–1.07)	0.067
rs10956697							
Co-dominant CC	86(37.9)	79(47.0)	1.00^ref^		16(44.4)	1.00^ref^	
AC	114(50.2)	70(41.7)	0.73(0.46–1.14)	0.165	18(50.0)	0.90(0.39–2.04)	0.792
AA	27(11.9)	19(11.3)	0.96(0.47–1.93)	0.902	2(5.6)	0.67(0.13–3.55)	0.636
Dominant CC	86(37.9)	79(47.0)	1.00^ref^		16(44.4)	1.00^ref^	
AC + AA	141(62.1)	89(53.0)	0.76(0.49–1.16)	0.201	20(55.6)	0.86(0.39–1.93)	0.719
Recessive CC + AC	200(88.1)	149(88.7)	1.00^ref^		34(94.4)	1.00^ref^	
AA	27(11.9)	19(11.3)	1.12(0.58–2.17)	0.739	2(5.6)	0.71(0.14–3.55)	0.677

*ref* reference; *AOR* Adjusted odds ratio; *95% CI* 95% confidence interval; adjusted for gender, age, BMI, ethnicity, smoking and drinking alcohol

When the subjects were further divided into subgroups according to the age, significant differences were found in the genotypic distributions of the WISP1 SNP rs10956697 in subjects ≥ 50 years old carrying the AC genotype as compared with those carrying the CC genotype (AC vs. CC: OR = 0.58, 95%CI 0.35–0.98, P = 0.043) (Additional file 1). No difference was observed in subjects < 50 years old.

As regards ethnicity, for the Han Chinese ethnicity, the presence of the rs7843546 T allele was associated with a significantly decreased risk of gastric cancer (CT vs. CC: OR = 0.33, 95%CI 0.14–0.78, P = 0.012; TT vs. CC: OR = 0.29, 95%CI 0.11–0.76, P = 0.012; dominant model CT + TT vs. CC: OR = 0.32, 95%CI 0.14–0.74, P = 0.007), whereas the association was not statistically significant among the Zhuang population cohort (Table 4).

In the smoking status cohort, compared with patients carrying the CC genotype of SNP rs10956697, those with the AC and AC + AA genotype exhibited a 0.28-fold lower risk and 0.32-fold lower risk of gastric cancer (OR = 0.28, 95% CI 0.09–0.82, P = 0.021 and OR = 0.32, 95% CI 0.12–0.89, P = 0.030, respectively) (Additional file 2). No difference was observed in the non-smoker cohort. No effect on the part of cell differentiation (Table 5), gender, or drinking status on the association between the WISP1 polymorphism and susceptibility to gastric cancer was observed. No significant association was observed between the WISP1 rs2929973 polymorphism and risk of gastric cancer in any genetic models.

## Discussion

WISP1 polymorphisms have been identified in various cancers, including breast cancer [13], urothelial cell carcinoma [14], hepatocellular carcinoma [15], oral squamous cell carcinoma [16], lung cancer [17], and uterine cervical cancer [18], but data are scant as to the involvement of WISP1 polymorphisms in gastric cancer. As far as we are aware, our study is the first to investigate the distributions of the rs2929973, rs7843546, and rs10956697 SNPs and their associations with the development of gastric cancer in a Guangxi Chinese population. Our results revealed the correlations between WISP1 SNPs (rs7843546 and rs10956697) and gastric cancer susceptibility in various subgroups. More specifically, the SNP rs7843546 TT and CT + TT genotype reduced susceptibility to stage I/II gastric cancer, with CC as a reference. The presence of the rs7843546 T allele was also associated with a significantly reduced risk of gastric cancer in a Han population. In addition, we found that smokers and subjects ≥ 50 years old carrying the AC or AC + AA genotype of the WISP1 rs10956697 polymorphism were less likely than CC homozygotes to develop gastric cancer. Both SNPs were discovered, for the first time, to be associated with the gastric cancer.

Most previous researches on the association between WISP1 polymorphisms and cancer were focused on the SNPs rs2929970, rs2929973, and rs2977530. The SNPs rs2929970 and rs2929973 are located in the 3′UTR of the WISP1 gene, and rs2977530 is located in introns. In the first second, in 2010, Frank et al*.* investigated the association between the WISP1 SNP rs2929970 and colorectal cancer risk but found no evidence for the said risk [26]. Then, in 2015, Chen et al*.* found that the WISP1 SNPs rs16893344, rs2977530, rs2977537, and rs62514004 were related to susceptibility to lung cancer but found no significant association for the SNPs rs2929970 or rs2929973 [23]. By contrast, Lau et al*.* demonstrated that WISP1 SNP rs2929970 carriers with at least one G allele were susceptible to oral squamous cell carcinoma in 2017 [17]. Moreover, Chen et al*.* revealed that the WISP1 SNP rs2977530 (AG + GG) was associated with hepatocellular carcinoma development and that WISP1 SNPs rs62514004 (AG + GG) and rs16893344 (CT + TT) were correlated with lower risks of large tumor size, reaching a later clinical stage of hepatocellular carcinoma, in 2018 [16]. Furthermore, Lin et al*.* demonstrated that the genotypes AG + GG in cases of WISP1 SNP rs2977530 reduced the susceptibility of Taiwanese women to invasive cervical cancer, whereas genotype AA in cases of rs2977537 increased said risk [19]. In addition, Lee et al*.* indicated that patients with urothelial cell carcinoma carrying rs2977530 genetic variants (AG + GG) had a higher risk of developing a more invasive tumor stage and a large tumor [15]. Wang et al*.* found that breast cancer patients with the WISP1 rs2929973 GG + TT genotype were likely to develop estrogen receptor (ER)- and progesterone receptor (PR)-positive tumor status [14]. Our study does not support the hypothesis that WISP1 polymorphisms contribute to gastric cancer risk. However, our results provide evidence that gastric cancer subsets may be affected. Our results parallel the previous results reported by Lee [15], Chen [16], and Frank [26] but are not in accord with the results reported by Lau [17], Lin [19], and Wang [14]. These results demonstrate the variety of WISP1 polymorphisms in various cancers. There are two potential reasons for these inconsistencies that we may consider. One is that WISP1 expression varies in different cancers. Recent research has revealed that the roles of WISP1 in cancer occurrence and progression are diverse in different kinds of cancer. For example, WISP1 was found to negatively regulate the progress of cell motility and invasion via the inhibition of Rac function through integrins in lung cancer [27]. In contrast, WISP-1 was up-regulated in gastric cancer tissues as compared with their adjacent noncancerous tissues, suggesting that WISP-1 acts as an oncogene in gastric cancer. Similar results were found in previous studies of liver cancer [28], breast cancer [29], and endometrial adenocarcinoma [30]. The other potential reason is that the various ethnicities of the patients included in the aforementioned studies. Frank [26] studied Caucasians, but Wang [14], Lin [19], Lee [15], Lau [17], and Chen [16, 18, 23] studied Asians. Our samples are taken from South Chinese population that is East Asian. In particular, our results revealed that the SNP rs7843546 TT genotype was associated with a significantly reduced risk of gastric cancer in a Han population but not a Zhuang population. This further indicated that WISP1 genotype distributions are different in different ethnicities.

*Helicobacter pylori* infection, aging, gender, smoking, and alcohol consumption are the main risk factor for the development of gastric cancer [31]. In order to rule out the influence of a confounding factor, *H. pylori* infection, we did not include *H. pylori* infection patients in our study. We further analyzed the correlations of WISP1 SNP polymorphisms with other confounding factors for gastric cancer patients. After stratifying individuals into smokers and nonsmokers, smokers with the AC genotype in WISP1 SNP rs10956697 displayed a 0.28-fold lower risk (95% CI 0.09–0.82) of gastric cancer. Smoking is a well-known carcinogen, including for gastric cancer, and nicotine exposure is suggested to promote cancer progression by activating the Wnt/β-catenin and Wnt/PCP signaling pathways [32]. Aging is also a significant risk factor for gastric cancer. We stratified our included subjects according to a person’s age. We found that subjects ≥ 50 years old carrying the WISP1 rs10956697 AC genotype were at a 0.58-fold (95% CI 0.35–0.98) lower risk than CC homozygotes of developing gastric cancer. Aging is the process of the degeneration of the body, from constitutive substances and tissue structures to physiological functions [33]. The time-dependent accumulation of cellular damage is widely considered to be the general cause of aging [34]. Concomitantly, cellular damage may occasionally provide aberrant advantages to certain cells, which can eventually produce cancer [33].

The current findings must be interpreted in light of several potential limitations. Firstly, we did not obtain positive results in overall cases, but we did obtain some positive results in the subgroup analysis. The evidence for different effects on the part of aging, smoking, and ethnicity on gastric cancer risk was suggestive but not conclusive. The sample size for the study was not large enough, and the sample size in each subgroup was too small; thus, the results lack statistical power and robustness. A larger independent-cohort study is required to confirm the result we obtained. Secondly, the study was limited to eligible participations in Guangxi (Southwest China), which may not be representative of the entire Chinese population. Therefore, these findings may not be generalized to other populations. Thirdly, this research was based on data from individual participants, and only three SNPs of the WISP1 gene were selected, which restricted interpretations regarding gene-to-gene interactions. These limitations restrict the interpretation and extrapolation of the current findings. The evidence for different effects on the part of age, smoking, ethnic group, and cancer stage on gastric cancer risk is suggestive but not conclusive. The mechanisms underlying these differences are also still unknown. One potential explanation may be the limited study numbers. After we stratified our population by age, smoking status, ethnic group, and cancer stage, the sample size grew smaller in each subgroup. Thus, the results lack statistical power and robustness. It would be interesting to identify more validation cohorts from other regions and more SNPs in the WISP1 gene and thus investigate their associations with gastric cancer risk.

## Conclusions

Overall, we could not identify a significant association between WISP1 SNPs rs2929973, rs7843546, and rs10956697 and gastric cancer risk. However, our results suggest that a subset of subjects may be affected, including patients with ≥ 50 years old carrying the AC genotype of rs10956697, smoking patients carrying the AC or AC + AA genotype of rs10956697, Han people carrying the CT or TT genotype of rs7843546, and stage I/II gastric patients carrying TT and CT + TT genotype of rs7843546. All these polymorphisms were, for the first time, discovered to represent a significantly reduced risk of gastric cancer. Replication in further epidemiologic studies and functional analyses is warranted to confirm these findings.

## Supplementary Information

**Additional file 1.** Distribution frequency of WISP1polymorphisms in controls and gastric cancer patients stratified by age.

**Additional file 2. **Distribution frequency of WISP1polymorphisms in controls and gastric cancer patients stratified by smoking status.

**Additional file 3. **Distribution frequency of WISP1polymorphisms in controls and gastric cancer patients stratified by gender.

**Additional file 4. **Distribution frequency of WISP1polymorphisms in controls and gastric cancer patients stratified by drinking status.

## Data Availability

The datasets supporting the conclusions of this article (are) included within the article and its additional files.

## Abbreviations

BMI: Body mass index
CI: Confidence interval
OR: Odds ratio
PCR-RFLP: Polymerase chain reaction-restriction fragment length polymorphism
SNP: Single-nucleotide polymorphism
WISP1: WNT1-inducible signaling pathway protein-1

## References

[1] Sung H, Ferlay J, Siegel RL (2021). Global cancer statistics 2020: GLOBOCAN estimates of incidence and mortality worldwide for 36 cancers in 185 countries. CA Cancer J Clin.

[2] International Agency for Research on Cancer. The global cancer observatory. Cancer today. http://gco.iarc.fr/. Accessed 8 Apr 2021.

[3] Klingelhöfer D, Braun M, Schöffel N, Brüggmann D, Groneberg DA (2020). Gastric cancer: bibliometric analysis of epidemiological, geographical and socio-economic parameters of the global research landscape. Int J Health Policy Manag.

[4] Plummer M, Franceschi S, Vignat J, Forman D, de Martel C (2015). Global burden of gastric cancer attributable to *Helicobacter pylori*. Int J Cancer.

[5] Hooi JKY, Lai WY, Ng WK, Suen MMY, Underwood FE, Tanyingoh D, Malfertheiner P, Graham DY, Wong VWS, Wu JCY (2017). Global prevalence of *Helicobacter pylori* infection: systematic review and meta-analysis. Gastroenterology.

[6] Kidd M, Lastovica AJ, Atherton JC, Louw JA (1999). Heterogeneity in the *Helicobacter pylori* vacA and cagA genes: association with gastroduodenal disease in South Africa?. Gut.

[7] Maiese K (2014). WISP1: clinical insights for a proliferative and restorative member of the CCN family. Curr Neurovasc Res.

[8] Berwick DC, Harvey K (2014). The regulation and deregulation of Wnt signaling by PARK genes in health and disease. J Mol Cell Biol.

[9] Gurbuz I, Chiquet-Ehrismann R (2015). CCN4/WISP1 (WNT1 inducible signaling pathway protein 1): a focus on its role in cancer. Int J Biochem Cell Biol.

[10] Jia S, Qu T, Feng M, Ji K, Li Z, Jiang W, Ji J (2017). Association of Wnt1-inducible signaling pathway protein-1 with the proliferation, migration and invasion in gastric cancer cells. Tumour Biol.

[11] Zhang LH, Wang Y, Fan QQ, Liu YK, Li LH, Qi XW, Mao Y, Hua D (2019). Up-regulated Wnt1-inducible signaling pathway protein 1 correlates with poor prognosis and drug resistance by reducing DNA repair in gastric cancer. World J Gastroenterol.

[12] Shastry BS (2009). SNPs: impact on gene function and phenotype. Methods Mol Biol.

[13] Davies SR, Watkins G, Mansel RE, Jiang WG (2007). Differential expression and prognostic implications of the CCN family members WISP-1, WISP-2, and WISP-3 in human breast cancer. Ann Surg Oncol.

[14] Wang Y, Yang SH, Hsu PW, Chien SY, Wang CQ, Su CM, Dong XF, Zhao YM, Tang CH (2019). Impact of WNT1-inducible signaling pathway protein-1 (WISP-1) genetic polymorphisms and clinical aspects of breast cancer. Medicine (Baltimore).

[15] Lee HL, Chiou HL, Wang SS, Hung SC, Chou MC, Yang SF, Hsieh MJ, Chou YE (2018). WISP1 genetic variants as predictors of tumor development with urothelial cell carcinoma. Urol Oncol.

[16] Chen CT, Lee HL, Chiou HL, Chou CH, Wang PH, Yang SF (2018). Impacts of WNT1-inducible signaling pathway protein 1 polymorphism on hepatocellular carcinoma development. PLoS ONE.

[17] Lau HK, Wu ER, Chen MK, Hsieh MJ, Yang SF, Wang LY, Chou YE (2017). Effect of genetic variation in microRNA binding site in WNT1-inducible signaling pathway protein 1 gene on oral squamous cell carcinoma susceptibility. PLoS ONE.

[18] Chen J, Yin J, Li X, Wang Y, Zheng Y, Qian C, Xiao L, Zou T, Wang Z, Liu J (2014). WISP1 polymorphisms contribute to platinum-based chemotherapy toxicity in lung cancer patients. Int J Mol Sci.

[19] Lin YH, Hsiao YH, Yang SF, Liu YF, Hsu CF, Wang PH (2018). Association between genetic polymorphisms of WNT1 inducible signaling pathway protein 1 and uterine cervical cancer. Reprod Sci.

[20] Gauderman WJ (2002). Sample size requirements for matched case-control studies of gene-environment interaction. Stat Med.

[21] Gauderman WJ (2002). Sample size requirements for association studies of gene-gene interaction. Am J Epidemiol.

[22] Li T, Qin W, Liu Y, Li S, Qin X, Liu Z (2017). Effect of RAGE gene polymorphisms and circulating sRAGE levels on susceptibility to gastric cancer: a case-control study. Cancer Cell Int.

[23] Chen J, Yin JY, Li XP, Wang Y, Zheng Y, Qian CY, He H, Fang C, Wang Z, Zhang Y et al. Association of Wnt-inducible signaling pathway protein 1 genetic polymorphisms with lung cancer susceptibility and platinum-based chemotherapy response. Clin Lung Cancer 2015, 16(4):298–304.e291–292.doi:10.1016/j.cllc.2014.12.008. 10.1016/j.cllc.2014.12.008 25656821

[24] Liu Y, Xie L, Zhao J, Huang X, Song L, Luo J, Ma L, Li S, Qin X (2015). Association between catalase gene polymorphisms and risk of chronic hepatitis B, hepatitis B virus-related liver cirrhosis and hepatocellular carcinoma in Guangxi population: a case-control study. Medicine (Baltimore).

[25] Nagai Y, Watanabe M, Ishikawa S, Karashima R, Kurashige J, Iwagami S, Iwatsuki M, Baba Y, Imamura Y, Hayashi N (2011). Clinical significance of Wnt-induced secreted protein-1 (WISP-1/CCN4) in esophageal squamous cell carcinoma. Anticancer Res.

[26] Frank B, Hoffmeister M, Klopp N, Illig T, Chang-Claude J, Brenner H (2010). Single nucleotide polymorphisms in Wnt signaling and cell death pathway genes and susceptibility to colorectal cancer. Carcinogenesis.

[27] Su F, Overholtzer M, Besser D, Levine AJ (2002). WISP-1 attenuates p53-mediated apoptosis in response to DNA damage through activation of the Akt kinase. Genes Dev.

[28] Calvisi DF, Conner EA, Ladu S, Lemmer ER, Factor VM, Thorgeirsson SS (2005). Activation of the canonical Wnt/beta-catenin pathway confers growth advantages in c-Myc/E2F1 transgenic mouse model of liver cancer. J Hepatol.

[29] Chiang KC, Yeh CN, Chung LC, Feng TH, Sun CC, Chen MF, Jan YY, Yeh TS, Chen SC, Juang HH (2015). WNT-1 inducible signaling pathway protein-1 enhances growth and tumorigenesis in human breast cancer. Sci Rep.

[30] Tang Q, Jiang X, Li H, Lin Z, Zhou X, Luo X, Liu L, Chen G (2011). Expression and prognostic value of WISP-1 in patients with endometrial endometrioid adenocarcinoma. J Obstet Gynaecol Res.

[31] Machlowska J, Baj J, Sitarz M, Maciejewski R, Sitarz R (2020). Gastric cancer: epidemiology, risk factors, classification, genomic characteristics and treatment strategies. Int J Mol Sci.

[32] Chung TT, Pan MS, Kuo CL, Wong RH, Lin CW, Chen MK, Yang SF (2011). Impact of RECK gene polymorphisms and environmental factors on oral cancer susceptibility and clinicopathologic characteristics in Taiwan. Carcinogenesis.

[33] López-Otín C, Blasco MA, Partridge L, Serrano M, Kroemer G (2013). The hallmarks of aging. Cell.

[34] Vijg J, Campisi J (2008). Puzzles, promises and a cure for ageing. Nature.

