# Supplementary material for: Association between WNT-1-inducible signaling pathway protein-1 (WISP1) genetic polymorphisms and the risk of gastric cancer in Guangxi Chinese
Source: Cancer Cell Int. 2021 Jul 30;21:405. doi: 10.1186/s12935-021-02116-2 (PMC8325280; doi:10.1186/s12935-021-02116-2)
Supplement: Supplementary file 1 — Additional file 1. Distribution frequency of WISP1polymorphisms in controls and gastric cancer patients stratified by age. [file 12935_2021_2116_MOESM1_ESM.docx]

Additional file 1. Distribution frequency of WISP1polymorphisms in controls and gastric cancer patients stratified by age

| Variables | Age <50 years | | | | Age ≥50 years | | | | |
| --- | --- | --- | --- | --- | --- | --- | --- | --- | --- |
|  | Cancer (N=72) | Controls (N=48) | AOR (95% CI) | *P* |  | Cancer (N=132) | Controls (N=179) | AOR (95% CI) | *P* |
| **rs2929973** | | | | | | |  | | |
| Co-dominant TT | 33 | 19 | 1.00^ref^ |  |  | 67 | 83 | 1.00^ref^ |  |
| TG | 32 | 19 | 0.91 (0.39-2.14) | 0.836 |  | 53 | 72 | 0.97 (0.57-1.64) | 0.906 |
| GG | 7 | 10 | 0.34 (0.12-1.29) | 0.122 |  | 12 | 24 | 0.66 (0.29-1.48) | 0.312 |
| Dominant TT | 33 | 19 | 1.00^ref^ |  |  | 67 | 83 | 1.00^ref^ |  |
| TG+GG | 39 | 29 | 0.72 (0.33-1.58) | 0.412 |  | 65 | 96 | 0.89 (0.54-1.45) | 0.632 |
| Recessive TT+TG | 65 | 38 | 1.00^ref^ |  |  | 120 | 155 | 1.00^ref^ |  |
| GG | 7 | 10 | 0.33 (0.09-1.22) | 0.096 |  | 12 | 24 | 0.67 (0.31-1.45) | 0.308 |
| **rs7843546** | |  |  |  |  |  |  |  |  |
| Co-dominant CC | 11 | 6 | 1.00^ref^ |  |  | 30 | 30 | 1.00^ref^ |  |
| CT | 37 | 28 | 0.80 (0.24-2.68) | 0.722 |  | 72 | 91 | 0.84 (0.45-1.59) | 0.592 |
| TT | 24 | 14 | 0.69 (0.19-2.49) | 0.575 |  | 30 | 58 | 0.61 (0.30-1.25) | 0.181 |
| Dominant CC | 11 | 6 | 1.00^ref^ |  |  | 30 | 30 | 1.00^ref^ |  |
| CT+TT | 61 | 42 | 0.77 (0.24-2.42) | 0.649 |  | 102 | 149 | 0.75 (0.41-1.38) | 0.356 |
| Recessive CT+CC | 48 | 34 | 1.00^ref^ |  |  | 102 | 121 | 1.00^ref^ |  |
| TT | 24 | 14 | 0.89 (0.33-2.35) | 0.808 |  | 30 | 58 | 0.70 (0.40-1.21) | 0.196 |
| **rs10956697** | | | | | | |  | | |
| Co-dominant CC | 27 | 20 | 1.00^ref^ |  |  | 68 | 19 | 1.00^ref^ |  |
| AC | 37 | 20 | 1.27 (0.54-2.99) | 0.578 |  | 51 | 94 | 0.58 (0.35-0.98) | **0.043** |
| AA | 8 | 8 | 0.75 (0.22-2.50) | 0.633 |  | 13 | 66 | 0.84 (0.36-1.96) | 0.679 |
| Dominant CC | 27 | 20 | 1.00^ref^ |  |  | 68 | 19 | 1.00^ref^ |  |
| AC+AA | 45 | 28 | 1.08 (0.49-2.40) | 0.845 |  | 64 | 160 | 0.62 (0.38-1.02) | 0.061 |
| Recessive CC+AC | 64 | 40 | 1.00^ref^ |  |  | 119 | 113 | 1.00^ref^ |  |
| AA | 8 | 8 | 0.60 (0.17-2.15) | 0.431 |  | 13 | 66 | 1.10 (0.49-2.48) | 0.816 |

ref: reference

AOR : Adjusted odds ratio; 95% CI, 95% confidence interval; adjusted for gender, BMI, ethnicity, smoking and drinking alcohol..
