# Supplementary material for: Association between WNT-1-inducible signaling pathway protein-1 (WISP1) genetic polymorphisms and the risk of gastric cancer in Guangxi Chinese
Source: Cancer Cell Int. 2021 Jul 30;21:405. doi: 10.1186/s12935-021-02116-2 (PMC8325280; doi:10.1186/s12935-021-02116-2)
Supplement: Supplementary file 2 — Additional file 2. Distribution frequency of WISP1polymorphisms in controls and gastric cancer patients stratified by smoking status. [file 12935_2021_2116_MOESM2_ESM.docx]

Additional file 2. Distribution frequency of WISP1polymorphisms in controls and gastric cancer patients stratified by smoking status

| Variables | Smokers | | | | Non-smokers | | | | |
| --- | --- | --- | --- | --- | --- | --- | --- | --- | --- |
|  | Cancer (N=58) | Controls (N=74) | AOR (95% CI) | *P* |  | Cancer (N=146) | Controls (N=156) | AOR (95% CI) | *P* |
| **rs2929973** | | | | | | |  | | |
| Co-dominant TT | 28 | 28 | 1.00^ref^ |  |  | 72 | 74 | 1.00^ref^ |  |
| TG | 22 | 30 | 0.62 (0.21-1.82) | 0.383 |  | 63 | 61 | 1.04 (0.62-1.73) | 0.881 |
| GG | 8 | 13 | 0.65 (0.16-2.60) | 0.543 |  | 11 | 21 | 0.50 (0.22-1.14) | 0.099 |
| Dominant TT | 28 | 28 | 1.00^ref^ |  |  | 72 | 74 | 1.00^ref^ |  |
| TG+GG | 30 | 43 | 0.63 (0.23-1.69) | 0.357 |  | 74 | 82 | 0.88 (0.54-1.42) | 0.600 |
| Recessive TT+TG | 50 | 58 | 1.00^ref^ |  |  | 135 | 135 | 1.00^ref^ |  |
| GG | 8 | 13 | 0.82 (0.23-2.95) | 0.756 |  | 11 | 21 | 0.48 (0.22-1.07) | 0.073 |
| **rs7843546** | |  |  |  |  |  |  |  |  |
| Co-dominant CC | 16 | 11 | 1.00^ref^ |  |  | 25 | 25 | 1.00^ref^ |  |
| CT | 28 | 38 | 0.63 (0.18-2.17) | 0.461 |  | 81 | 81 | 1.12 (0.57-2.19) | 0.738 |
| TT | 14 | 22 | 0.48 (0.13-1.79) | 0.274 |  | 40 | 50 | 0.87 (0.42-1.80) | 0.701 |
| Dominant CC | 16 | 11 | 1.00^ref^ |  |  | 25 | 25 | 1.00^ref^ |  |
| CT+TT | 42 | 60 | 0.56 (0.18-1.76) | 0.320 |  | 121 | 131 | 1.03 (0.54-1.95) | 0.927 |
| Recessive CT+CC | 4.00 | 49.00 | 1.00ref |  |  | 106 | 106 | 1.00ref |  |
| TT | 14 | 22 | 0.64 (0.22-1.84) | 0.406 |  | 40 | 50 | 0.82 (0.48-1.38) | 0.447 |
| **rs10956697** | | | | | | |  | | |
| Co-dominant CC | 32 | 22 | 1.00^ref^ |  |  | 63 | 64 | 1.00^ref^ |  |
| AC | 19 | 38 | 0.28 (0.09-0.82) | **0.021** |  | 69 | 76 | 0.95 (0.57-1.58) | 0.850 |
| AA | 7 | 11 | 0.51 (0.11-2.34) | 0.385 |  | 14 | 16 | 0.87 (0.38-2.02) | 0.746 |
| Dominant CC | 32 | 22 | 1.00^ref^ |  |  | 63 | 64 | 1.00^ref^ |  |
| AC+AA | 26 | 49 | 0.32 (0.12-0.89) | **0.030** |  | 83 | 92 | 0.92 (0.56-1.50) | 0.731 |
| Recessive CC+AC | 51 | 60 | 1.00^ref^ |  |  | 132 | 140 | 1.00^ref^ |  |
| AA | 7 | 11 | 0.97 (0.24-3.95) | 0.969 |  | 14 | 16 | 0.88 (0.40-1.95) | 0.756 |

ref: reference

AOR : Adjusted odds ratio; 95% CI, 95% confidence interval; adjusted for gender, age, BMI, ethnicity and drinking alcohol.
