# Supplementary material for: Association between WNT-1-inducible signaling pathway protein-1 (WISP1) genetic polymorphisms and the risk of gastric cancer in Guangxi Chinese
Source: Cancer Cell Int. 2021 Jul 30;21:405. doi: 10.1186/s12935-021-02116-2 (PMC8325280; doi:10.1186/s12935-021-02116-2)
Supplement: Supplementary file 3 — Additional file 3. Distribution frequency of WISP1polymorphisms in controls and gastric cancer patients stratified by gender. [file 12935_2021_2116_MOESM3_ESM.docx]

Additional file 3. Distribution frequency of WISP1polymorphisms in controls and gastric cancer patients stratified by gender

| Variables | Males | | | | Females | | | | |
| --- | --- | --- | --- | --- | --- | --- | --- | --- | --- |
|  | Cancer (N=134) | Controls (N=168) | AOR (95% CI) | *P* |  | Cancer (N=70) | Controls (N=119) | AOR (95% CI) | *P* |
| **rs2929973** | | | | | | |  | | |
| Co-dominant TT | 69 | 49 | 1.00^ref^ |  |  | 31 | 53 | 1.00^ref^ |  |
| TG | 52 | 43 | 0.86 (0.48-1.55) | 0.612 |  | 33 | 48 | 1.33 (0.59-3.00) | 0.486 |
| GG | 13 | 16 | 0.56 (0.24-1.33) | 0.191 |  | 6 | 18 | 0.51 (0.15-1.78) | 0.292 |
| Dominant TT | 69 | 49 | 1.00^ref^ |  |  | 31 | 53 | 1.00^ref^ |  |
| TG+GG | 65 | 59 | 0.75 (0.44-1.30) | 0.311 |  | 39 | 66 | 1.09 (0.51-2.31) | 0.833 |
| Recessive TT+TG | 121 | 92 | 1.00^ref^ |  |  | 64 | 101 | 1.00^ref^ |  |
| GG | 13 | 16 | 0.59 (0.26-1.35) | 0.212 |  | 6 | 18 | 0.45 (0.14-1.46) | 0.182 |
| **rs7843546** | |  |  |  |  |  |  |  |  |
| Co-dominant CC | 28 | 20 | 1.00^ref^ |  |  | 13 | 16 | 1.00^ref^ |  |
| CT | 71 | 53 | 0.98 (0.48-2.00) | 0.961 |  | 38 | 66 | 1.19 (0.42-3.32) | 0.745 |
| TT | 35 | 35 | 0.77 (0.35-1.68) | 0.513 |  | 19 | 37 | 0.81 (0.26-2.50) | 0.708 |
| Dominant CC | 28 | 20 | 1.00^ref^ |  |  | 13 | 16 | 1.00^ref^ |  |
| CT+TT | 106 | 88 | 0.91 (0.47-1.78) | 0.783 |  | 57 | 103 | 1.03 (0.39-2.74) | 0.947 |
| Recessive CT+CC | 99 | 73 | 1.00^ref^ |  |  | 51 | 82 | 1.00^ref^ |  |
| TT | 35 | 35 | 0.82 (0.45-1.47) | 0.497 |  | 19 | 37 | 0.71 (0.30-1.66) | 0.431 |
| **rs10956697** | | | | | | |  | | |
| Co-dominant CC | 67 | 46 | 1.00^ref^ |  |  | 28 | 40 | 1.00^ref^ |  |
| AC | 53 | 49 | 0.76 (0.43-1.35) | 0.347 |  | 35 | 65 | 0.81 (0.36-1.82) | 0.605 |
| AA | 14 | 13 | 0.80 (0.33-1.95) | 0.622 |  | 7 | 14 | 0.89 (0.24-3.35) | 0.862 |
| Dominant CC | 67 | 46 | 1.00^ref^ |  |  | 28 | 40 | 1.00^ref^ |  |
| AC+AA | 67 | 62 | 0.75 (0.43-1.28) | 0.289 |  | 42 | 79 | 0.82 (0.38-1.80) | 0.620 |
| Recessive CC+AC | 120 | 95 | 1.00^ref^ |  |  | 63 | 105 | 1.00^ref^ |  |
| AA | 14 | 13 | 0.90 (0.39-2.08) | 0.802 |  | 7 | 14 | 1.01 (0.29-3.48) | 0.991 |

ref: reference

AOR : Adjusted odds ratio; 95% CI, 95% confidence interval; adjusted for age, BMI, ethnicity, smoking and drinking alcohol..
