# Supplementary material for: Association between WNT-1-inducible signaling pathway protein-1 (WISP1) genetic polymorphisms and the risk of gastric cancer in Guangxi Chinese
Source: Cancer Cell Int. 2021 Jul 30;21:405. doi: 10.1186/s12935-021-02116-2 (PMC8325280; doi:10.1186/s12935-021-02116-2)
Supplement: Supplementary file 4 — Additional file 4. Distribution frequency of WISP1polymorphisms in controls and gastric cancer patients stratified by drinking status. [file 12935_2021_2116_MOESM4_ESM.docx]

Additional file 4. Distribution frequency of WISP1polymorphisms in controls and gastric cancer patients stratified by drinking status

| Variables | Drinkers | | | | Non-drinkers | | | | |
| --- | --- | --- | --- | --- | --- | --- | --- | --- | --- |
|  | Cancer (N=52) | Controls (N=67) | AOR (95% CI) | *P* |  | Cancer (N=152) | Controls (N=160) | AOR (95% CI) | *P* |
| **rs2929973** | | | | | | |  | | |
| Co-dominant TT | 27 | 32 | 1.00^ref^ |  |  | 73 | 70 | 1.00^ref^ |  |
| TG | 18 | 25 | 0.63 (0.21-1.94) | 0.422 |  | 67 | 66 | 1.07 (0.65-1.78) | 0.790 |
| GG | 7 | 10 | 0.74 (0.17-3.29) | 0.692 |  | 12 | 24 | 0.50 (0.22-1.11) | 0.087 |
| Dominant TT | 27 | 32 | 1.00^ref^ |  |  | 73 | 70 | 1.00^ref^ |  |
| TG+GG | 25 | 35 | 0.66 (0.25-1.80) | 0.421 |  | 79 | 90 | 0.90 (0.56-1.44) | 0.650 |
| Recessive TT+TG | 45 | 57 | 1.00^ref^ |  |  | 140 | 136 | 1.00^ref^ |  |
| GG | 7 | 10 | 0.86 (0.21-3.62) | 0.841 |  | 12 | 24 | 0.48 (0.22-1.02) | 0.057 |
| **rs7843546** | |  |  |  |  |  |  |  |  |
| Co-dominant CC | 12 | 13 | 1.00^ref^ |  |  | 29 | 23 | 1.00^ref^ |  |
| CT | 28 | 30 | 1.47 (0.41-5.32) | 0.554 |  | 81 | 89 | 0.81 (0.42-1.57) | 0.538 |
| TT | 12 | 24 | 0.54 (0.14-2.06) | 0.366 |  | 42 | 48 | 0.71 (0.34-1.45) | 0.342 |
| Dominant CC | 12 | 13 | 1.00^ref^ |  |  | 29 | 23 | 1.00^ref^ |  |
| CT+TT | 40 | 54 | 0.95 (0.30-3.02) | 0.931 |  | 123 | 137 | 0.78 (0.42-1.46) | 0.434 |
| Recessive CT+CC | 40 | 43 | 1.00^ref^ |  |  | 110 | 112 | 1.00^ref^ |  |
| TT | 12 | 24 | 0.42 (0.14-1.26) | 0.121 |  | 42 | 48 | 0.85 (0.50-1.42) | 0.528 |
| **rs10956697** | | | | | | |  | | |
| Co-dominant CC | 26 | 20 | 1.00^ref^ |  |  | 69 | 66 | 1.00^ref^ |  |
| AC | 20 | 38 | 0.49 (0.17-1.40) | 0.182 |  | 68 | 76 | 0.88 (0.53-1.45) | 0.608 |
| AA | 6 | 9 | 0.62 (0.12-3.19) | 0.568 |  | 15 | 18 | 0.88 (0.39-2.00) | 0.765 |
| Dominant CC | 26 | 20 | 1.00^ref^ |  |  | 69 | 66 | 1.00^ref^ |  |
| AC+AA | 26 | 47 | 0.51 (0.19-1.40) | 0.191 |  | 83 | 94 | 0.86 (0.53-1.39) | 0.541 |
| Recessive CC+AC | 46 | 58 | 1.00^ref^ |  |  | 137 | 142 | 1.00^ref^ |  |
| AA | 6 | 9 | 0.89 (0.19-4.13) | 0.877 |  | 15 | 18 | 0.93 (0.43-2.03) | 0.860 |

ref: reference

AOR : Adjusted odds ratio; 95% CI, 95% confidence interval; adjusted for gender, age, BMI, ethnicity and smoking.
